# Chronic kidney disease and incident cancer risk: an individual participant data meta-analysis

**DOI:** 10.1038/s41416-025-03140-z

**Published:** 2025-09-06

**Authors:** Yejin Mok, Aditya Surapaneni, Yingying Sang, Josef Coresh, Morgan E. Grams, Kunihiro Matsushita, Shoshana H. Ballew, Natalia Alencar de Pinho, Johan Ärnlöv, Sandhi M. Barreto, Samira Bell, Hermann Brenner, Juan-Jesus Carrero, Rajkumar Chinnadurai, Elizabeth Ciemins, Ron T. Gansevoort, Simerjot K. Jassal, Keum Ji Jung, H. Lester Kirchner, Tsuneo Konta, Csaba P. Kovesdy, Li Luo, Krutika Pandit, Mahboob Rahman, Cassianne Robinson-Cohen, Charumathi Sabanayagam, Ulla T. Schultheiss, Michael Shlipak, Natalie Staplin, Marcello Tonelli, Angela Yee-Moon Wang, Chi-Pang Wen, Mark Woodward, Jennifer S. Lees

**Affiliations:** 1 Department of Epidemiology, Johns Hopkins Bloomberg School of Public Health, and Welch Center for Prevention, Epidemiologyand Clinical Research, Baltimore, MD USA; 2 Division of Precision Medicine, Department of Medicine, New York University Grossman School of Medicine, New York, NY USA; 3 Optimal Aging Institute, New York University Grossman School of Medicine, New York, NY USA; 4 Centre for research in Epidemiology and Population Health (CESP), Paris-Saclay University, Inserm U1018, Versailles Saint-Quentin University, Clinical Epidemiology Team, Villejuif, France; 5 Department of Neurobiology, Care Sciences and Society, Division of Family Medicine and Primary Care, Karolinska Institutet, Huddinge, Sweden; 6 School of Health and Social Studies, Dalarna University, Falun, Sweden; 7 Department of Preventive Medicine, School of Medicine and Hospital das Clínicas/Empresa Brasileira de Serviços Hospitalares and Universidade Federal de Minas Gerais, Belo Horizonte, Brazil; 8 Division of Population Health and Genomics, School of Medicine, University of Dundee, Dundee, UK; 9 Division of Clinical Epidemiology and Aging Research, German Cancer Research Center, and Network Aging Research, Heidelberg University, Heidelberg, Germany; 10 Department of Medical Epidemiology and Biostatistics, Karolinska Institutet, Huddinge, Sweden; 11 Division of Nephrology, Department of Clinical Sciences, Karolinska Institutet, Danderyd Hospital, Stockholm, Sweden; 12 Department of Renal Medicine, Salford Care Organisation, Northern Care Alliance NHS Foundation Trust, Salford, UK; 13 AMGA (American Medical Group Association), Alexandria, VA USA; 14 Department of Nephrology, University Medical Center Groningen, University of Groningen, Groningen, Netherlands; 15 University of California San Diego, La Jolla, CA USA; 16 San Diego VA Health Care System, San Diego, CA USA; 17 Institute for Health Promotion, Department of Epidemiology and Health Promotion, Graduate School of Public Health, Yonsei University, Seoul, Republic of Korea; 18 Department of Population Health Sciences, Research Institute, Geisinger, Danville, PA USA; 19 Department of Public Health and Hygiene, Yamagata University Faculty of Medicine, Yamagata, Japan; 20 Medicine-Nephrology, Memphis Veterans Affairs Medical Center and University of Tennessee Health Science Center, Memphis, TN USA; 21 Division of Nephrology and Hypertension, University Hospitals Cleveland Medical Center, Louis Stokes Cleveland Veterans Affairs Medical Center, Case Western Reserve University, Cleveland, OH USA; 22 Division of Nephrology and Hypertension, Department of Medicine, Vanderbilt University Medical Center, Nashville, TN USA; 23 Singapore Eye Research Institute, Singapore National Eye Centre, Singapore, Singapore; 24 Ophthalmology and Visual Sciences Academic Clinical Programme (EYE-ACP), Duke-NUS Medical School, Singapore, Singapore; 25 Institute of Genetic Epidemiology, Department of Data Driven Medicine, Faculty of Medicine and Medical Center, University of Freiburg, Freiburg, Germany; 26 Department of Medicine IV, Nephrology and Primary Care, Faculty of Medicine and Medical Center - University of Freiburg, Freiburg, Germany; 27 Synlab, MVZ Humangenetik Freiburg GmbH, Freiburg, Germany; 28 Kidney Health Research Collaborative, Department of Medicine, University of California San Francisco, San Francisco, CA USA; 29 General Internal Medicine Division, Medical Service, San Francisco Veterans Affairs Health Care System, San Francisco, CA USA; 30 Renal Studies Group, Clinical Trial Service Unit and Epidemiological Studies Unit, Nuffield Department of Population Health, University of Oxford, Oxford, UK; 31 Department of Medicine, University of Calgary, Calgary, AB Canada; 32 Department of Renal Medicine, Singapore General Hospital, Duke-National University of Singapore, Singapore, Singapore; 33 Institute of Population Health Science, National Health Research Institutes/China Medical University Hospital, Zhunan, Taiwan; 34 The George Institute for Global Health, University of New South Wales, Sydney, Australia; 35 The George Institute for Global Health, Imperial College London, London, UK; 36 School of Cardiovascular and Metabolic Health, University of Glasgow, Glasgow, UK; 37 Glasgow Renal and Transplant Unit, Queen Elizabeth University Hospital, Glasgow, UK; 38 Department of Medicine, Kidney Health Research Group, University of Alberta, Edmonton, Canada; 39 Public Health Department, Epidémiologie - Maison régionale de la recherche clinique, CHU Lille, Lille, France; 40 UMR1167 RIDAGE, Institut Pasteur de Lille, INSERM, Univ Lille, Lille, France; 41 Division of Nephrology, University of Michigan, Ann Arbor, MI USA; 42 University of New Mexico Health Sciences Center, Albuquerque, NM USA; 43 Division of Biostatistics, University of Pennsylvania, Philadelphia, PA USA; 44 Universidade Federal do Rio Grande do Sul, Porto Alegre, 90035-003 Rio Grande do Sul Brazil; 45 Federal University of Espírito Santo, Vitória, Espírito Santo Brazil; 46 Universidade de São Paulo, São Paulo, São Paulo estado Brazil; 47 Ulm University, Ulm, Germany; 48 University Hospital Erlangen, Friedrich-Alexander-Universität Erlangen-Nürnberg, Erlangen, Germany; 49 Faculty of Medicine and Medical Center - University of Freiburg, Freiburg, Germany; 50 Charité-Universitätsmedizin Berlin, Berlin, Germany; 51 University of Washington, Seattle, WA USA; 52 University Medical Center Groningen, University of Groningen, Groningen, Netherlands; 53 Erasmus MC, Cardiovascular Institute, Thorax Center, Department of Cardiology, Rotterdam, The Netherlands; 54 University of California San Diego, San Diego, CA USA; 55 University of Tennessee Health Science Center, Memphis, TN USA; 56 Uppsala University, Uppsala, Sweden; 57 Tufts Medical Center, Boston, MA USA; 58 London School of Hygiene and Tropical Medicine, London, UK

**Keywords:** Risk factors, Predictive markers, Cancer epidemiology

## Abstract

**Background:**

Studies examining the association of chronic kidney disease (CKD) with cancer risk have demonstrated conflicting results.

**Methods:**

This was an individual participant data meta-analysis including 54 international cohorts contributing to the CKD Prognosis Consortium. Included cohorts had data on albuminuria [urine albumin-to-creatinine ratio (ACR)], estimated glomerular filtration rate (eGFR), overall and site-specific cancer incidence, and established risk factors for cancer. Included participants were aged 18 years or older, without previous cancer or kidney failure.

**Results:**

Among 1,319,308 individuals, the incidence rate of overall cancer was 17.3 per 1000 person-years. Higher ACR was positively associated with cancer risk [adjusted hazard ratio 1.08 (95% CI 1.06–1.10) per 8-fold increase in ACR]. No association of eGFR with overall cancer risk was seen. For site-specific cancers, lower eGFR was associated with urological cancer and multiple myeloma, whereas higher ACR was associated with many cancer types (kidney, head/neck, colorectal, liver, pancreas, bile duct, stomach, larynx, lung, hemolymphatic, leukaemia, and multiple myeloma). Results were similar in a 1-year landmark analysis.

**Discussion:**

Albuminuria, but not necessarily eGFR, was independently associated with the subsequent risk of cancer. Our results warrant an investigation into mechanisms that explain the link between albuminuria and cancer.

## Introduction

Among people with chronic kidney disease (CKD), cancer has been shown to be one of the leading causes of death [1, 2]. Across global populations, survival after a diagnosis of cancer has consistently been shown to be lower among people with CKD [2–19]. However, it is not clear whether non-dialysis CKD is associated with an increased cancer incidence: a finding that may warrant exploration into tailored screening and diagnostic pathways for cancer among people with CKD.

Data on the two most commonly measured kidney measures, estimated glomerular filtration rate (eGFR; a marker of kidney filtration) and albuminuria (as a marker of glomerular damage), often demonstrate different results for each component. Many studies show associations between eGFR and cancer types where reverse causality might exist, such as kidney and urothelial cancer [2–4, 6, 8, 14, 17, 19], but a null association of low eGFR with overall cancer risk [5, 12, 13, 15, 16]. In contrast, most studies investigating albuminuria showed a modest association of albuminuria with cancer risk, independent of eGFR and diabetes [5–7, 9, 10, 16, 18]. Particularly, albuminuria has been consistently associated with lung cancer, in addition to kidney-related cancer [5, 6, 9, 10, 16]. Most previous studies included only one of eGFR or albuminuria [2–4, 8, 11, 14, 15], and few have evaluated the association with eGFR based on creatinine and cystatin C.

Therefore, we quantified the independent associations of albuminuria and eGFR with the incidence of overall cancer, and among selected site-specific cancers, using data from eligible cohorts in the Chronic Kidney Disease Prognosis Consortium (CKD-PC).

## Methods

### Study design and data source

The CKD-PC is an international consortium established to provide evidence that can improve the prevention and management of CKD. It currently consists of more than 110 prospective cohorts, including >30 million participants from over 40 countries with data for eGFR, albuminuria, phenotypes, and clinical outcomes. The current study included the 54 cohorts that had data on serum creatinine, albuminuria, incident cancer risk, and established risk factors for cancer (i.e., body mass index [BMI] and smoking). Within these cohorts, the study population was restricted to participants aged 18 years or older without a history of cancer or end-stage kidney disease requiring maintenance kidney replacement therapy. This study was approved for use of de-identified or limited data by the institutional review board at New York University.

### Kidney measures

Albuminuria was estimated using the urine albumin-to-creatinine ratio (ACR) or the urine protein creatinine ratio (PCR), available in 50 cohorts. Both spot and 24-hour collections were accepted, and the protein-to-creatinine values were converted to the urine ACR using a previously published conversion equation [20]. We also used semi-quantitative dipstick proteinuria as a secondary albuminuria measure, available in 37 cohorts. In research cohorts, ACR and eGFR were assessed at the same time point. In real-world cohorts, we set the baseline as the first eGFR measurement after one year in the health system and ACR, PCR, and dipstick were the nearest values prior to baseline and within 3 years.

For the estimation of kidney function, we assessed the association between eGFR and cancer using two equations and biomarkers. Creatinine is the most used biomarker of kidney function in clinical practice. Cystatin C is less commonly available, but in combination with creatinine, improves the accuracy of kidney function estimation compared to creatinine alone [21]. Therefore, in the primary analysis, we used eGFR using the Chronic Kidney Disease Epidemiology Collaboration (CKD-EPI) creatinine-based equation (eGFRcr) [22]. As a secondary analysis, we analysed eGFR using the CKD-EPI cystatin C and creatinine equation (eGFRcr-cys) in the 8 cohorts with available cystatin C.

### Outcomes

The outcomes of interest were overall and site-specific cancer incidence. Some cohorts performed expert adjudication for cancer, some were linked to the cancer registry, and some identified outcomes based on coding alone using the International Classification of Diseases codes (**Appendix 1**). Cohorts with fewer than 50 events for cancer outcomes were excluded from the meta-analysis.

### Covariates

History of cardiovascular disease (CVD) was defined as previous myocardial infarction, bypass grafting, percutaneous coronary intervention, heart failure, or stroke. Hypertension was defined as systolic blood pressure/diastolic blood pressure ≥140/≥90 mmHg or use of antihypertensive medication. Diabetes was defined as fasting/non-fasting glucose ≥7.0/≥11.1 mmol/L, use of glucose-lowering drugs, or self-reported physician diagnosis of diabetes. Smoking status was categorized as current, former or never smoking. Total cholesterol and body mass index (BMI) were defined as the nearest outpatient values to baseline and within 3 years.

### Statistical analysis

Three study populations were used. For the primary analysis, only participants with ACR or converted protein-creatinine values were included (“ACR population”; 50 cohorts; *N* = 1,319,308). For the secondary analysis, we included participants with dipstick values (“dipstick population”; 37 cohorts; 3,532,279; participants with both ACR or PCR and dipstick measures available were included in both the ACR population and dipstick populations). In the third analysis, we included participants with measures of creatinine, cystatin, and ACR or converted protein-creatinine values (“eGFRcr-cys population”; 8 cohorts; *N* = 453,628). Baseline characteristics were summarized within cohorts and then overall.

In sensitivity analyses within the Optum Labs Data Warehouse (OLDW), a longitudinal, real-world data asset with de-identified administrative claims and electronic health record (EHR) data, we observed only modest attenuation of associations of ACR and eGFR with cancer risk between unadjusted, age-sex adjusted, and fully-adjusted models. Therefore, in the ACR population (50 participating cohorts), we first quantified the association of albuminuria and eGFRcr with overall cancer risk after adjusting for each other and potential confounders (age, sex, BMI, hypertension, diabetes, total cholesterol, use of aspirin or glucocorticoids/other immunosuppressant, history of CVD, and smoking status [never vs. former vs. current]) using Cox proportional hazards models censored at death or last encounter. In the categorical analyses, individuals were classified by ACR (<10, 10–29, 30–299, and ≥300 mg/g) and eGFRcr (<15, 15–29, 30–44, 45–59, 60–89, 90–104, and ≥105 mL/min/1.73m^2^) categories. For continuous analyses, we modelled eGFRcr and log-transformed ACR using linear splines with knots at ACR 10, 30, and 300 mg/g and eGFRcr 30, 45, 60, 75, 90, and 105 mL/min/1.73m^2^. We also quantified the risk of overall cancer by cross-categories of ACR and eGFRcr.

We conducted several sensitivity analyses. First, models were run by stratum for demographic and clinical subgroups: age (<65 years versus ≥65 years), sex (female vs. male), smoking status (never vs. former vs. current), BMI category (<25 vs. 25–29 vs. 30+ kg/m^2^), diabetes, hypertension, aspirin use, history of CVD and eGFRcr (<60 vs. 60+ mL/min/1.73m^2^). For this analysis, we modelled kidney measures as continuous (per 8-fold increment in ACR and per 15 mL/min/1.73m^2^ decrement in eGFRcr with a knot at 60 mL/min/1.73m^2^). Second, using the dipstick population, we estimated the associations with cancer for dipstick proteinuria levels (negative, trace, and positive [1+, 2+, and 3+]) using the same covariates as described and modelling eGFRcr as in the categorical analyses. Third, we included 8 cohorts with the data on eGFRcr-cys and quantified the association of eGFRcr-cys with cancer risk. Fourth, we repeated analyses starting follow-up 1 year after baseline (i.e., one year of lag time) to reduce the possibility of reverse causation. Fifth, we adjusted for multiple testing for site-specific cancers using the Bonferroni correction. Sixth, we repeated the primary analysis according to the primary population type in the contributing cohort: high CKD, high CVD, research, and EHR cohorts.

Statistics were first obtained within each cohort and then pooled by an inverse-variance weighted random-effects model. All analyses were conducted using Stata MP version 18 (StataCorp). Statistical significance was determined by a 2-sided *P* < 0.05.

## Results

### Study population characteristics

There were 50 cohorts, including 1,319,308 individuals, with data available for eGFRcr and ACR or protein-to-creatinine ratio and included in the analyses (Table 1 and Table S1). Among these individuals, the mean age was 57 years, 47% were female, the mean BMI was 30 kg/m^2^, 44% had diabetes (reflecting cohorts selected for ACR assessment), the median ACR was 11 mg/g, and the mean eGFRcr was 88 mL/min/1.73 m^2^. There were 37 cohorts with dipstick proteinuria including 3,532,279 individuals, and the mean age was 47 years, 60% were female, the mean BMI was 29 kg/m^2^, 9% had diabetes, 81.3% had negative dipstick protein, 9.2% had trace, 6.1% 1+, 2.5% 2+, and 0.9% 3+, and the mean eGFRcr was 94 mL/min/1.73 m^2^ (Table 1 and Table S2). There were 8 cohorts with cystatin C available, including 453,628 individuals, and the mean age was 57 years, 53% were female, the mean BMI was 28 kg/m^2^, 6% had diabetes, the median ACR was 6 mg/g, and mean eGFRcr-cys was 94 mL/min/1.73 m^2^ (Table 1 and Table S3).

**Table 1:** Participants characteristics for the three study populations.

	ACR population	Dipstick population	eGFRcr-cys population
No of cohorts	50	37	8
No of individuals	1,319,308	3,532,279	453,628
Age, y (SD)	57.1 (12.3)	46.5 (16.2)	56.5 (8.2)
Women	625,329 (47.4)	2,107,094 (59.7)	240,112 (52.9)
Body mass index, kg/m^2^ (SD)	30.4 (7.8)	28.6 (6.6)	27.5 (4.8)
Total cholesterol, mmol/L (SD)	5.0 (1.0)	4.8 (0.9)	5.7 (1.1)
Diabetes	574,422 (43.5)	334,670 (9.5)	28,714 (6.3)
Hypertension	704,126 (53.4)	971,546 (27.5)	131,694 (29.0)
Aspirin use	130,353 (10.1)	63,752 (2.1)	66,480 (14.9)
Glucocorticoid/other immunosuppressant use	29,861 (2.4)	123,549 (4.0)	3435 (0.8)
Smoking status			
Never	914,097 (69.3)	2,961,556 (83.8)	246,242 (54.3)
Former	285,922 (21.7)	298,812 (8.5)	157,214 (34.7)
Current	119,289 (9.0)	271,912 (7.7)	50,172 (11.1)
History of CVD	188,481 (14.3)	242,135 (6.9)	29,173 (6.4)
eGFRcr, mL/min/1.73 m^2^ (SD)	88.0 (19.8)	94.3 (21.2)	93.7 (13.2)
eGFRcr-cys, mL/min/1.73 m^2^ (SD)	—	—	94.0 (14.6)
ACR, mg/g [IQI]	11.0 [6.0–13.0]		6.0 [6.0–6.0]
Dipstick proteinuria			
Negative	—	2,872,898 (81.3)	—
Trace	—	326,360 (9.2)	—
+1	—	214,900 (6.1)	—
+2	—	87,306 (2.5)	—
+3	—	30,817 (0.9)	—

Numbers provided are *n* (%) except where indicated. *SD* standard deviation, *IQI* inter-quartile interval. Columns are not mutually exclusive: an individual could hypothetically be included in all three if they had a measure of ACR, a measure of dipstick protein, and an eGFRcr-cys.

The incidence rate of overall cancer was 17 events per 1000 person-years (15 and 19 events per 1000 person-years for females and males, respectively) (Table 2). The incidence rates per 1000 person-years of site-specific cancer were lowest at 0.05 for gallbladder cancer and highest at 4.9 and 4.2 for sex-specific cancers, prostate cancer, and breast cancer, respectively.

**Table 2:** Crude incidence rate (IR) per 1000 person-years (95% CI) of cancer by sex in the ACR population^a^.

Outcome	Both sexes			Female			Male		
	*N*	Events	IR	*N*	Events	IR	*N*	Events	IR
Overall cancer	1,317,796	153,236 (11.6%)	17.27 (15.56–23.57)	624,416	63,736 (10.2%)	15.40 (12.98–23.21)	693,380	89,500 (12.9%)	19.33 (16.81–22.88)
Site-specific cancer									
Bileduct	1,265,236	727 (0.1%)	0.09 (0.05–0.13)	599,350	268 (0.0%)	0.07 (0.04–0.10)	665,886	459 (0.1%)	0.12 (0.06–0.15)
Bladder	1,286,526	7390 (0.6%)	0.83 (0.61–1.08)	609,412	1255 (0.2%)	0.33 (0.18–0.49)	677,114	6135 (0.9%)	1.28 (1.00–1.65)
Brain	1,287,960	2141 (0.2%)	0.22 (0.13–0.29)	610,967	804 (0.1%)	0.18 (0.09–0.28)	676,993	1337 (0.2%)	0.23 (0.14–0.31)
Breast	615,901	16976 (2.8%)	4.17 (3.44–4.95)	615,901	16976 (2.8%)	4.17 (3.44–4.95)			
Cervical	604,771	729 (0.1%)	0.26 (0.17–0.37)	604,771	729 (0.1%)	0.26 (0.17–0.37)			
Colon	1,278,280	9637 (0.8%)	1.10 (0.90–1.32)	604,904	3771 (0.6%)	0.98 (0.73–1.32)	673,376	5866 (0.9%)	1.20 (1.03–1.38)
Colorectal	1,270,159	12135 (1.0%)	1.35 (1.18–1.56)	602,787	4759 (0.8%)	1.19 (0.94–1.54)	667,372	7376 (1.1%)	1.44 (1.27–1.64)
Gallbladder	1,265,236	309 (0.0%)	0.04 (0.02–0.06)	599,350	167 (0.0%)	0.04 (0.00–0.07)	665,886	142 (0.0%)	0.02 (0.00–0.05)
Head neck	1,260,924	3966 (0.3%)	0.42 (0.30–0.59)	595,823	1052 (0.2%)	0.23 (0.17–0.36)	665,101	2914 (0.4%)	0.56 (0.40–0.75)
Hemolymphatic	1,276,030	11384 (0.9%)	1.53 (1.18–1.88)	603,756	4219 (0.7%)	1.37 (0.85–1.62)	672,274	7165 (1.1%)	1.70 (1.26–2.24)
Kidney	1,288,259	5732 (0.4%)	0.82 (0.67–0.97)	610,349	1733 (0.3%)	0.61 (0.44–0.84)	677,910	3999 (0.6%)	1.00 (0.81–1.27)
Larynx	1,265,236	932 (0.1%)	0.10 (0.05–0.15)	599,350	128 (0.0%)	0.04 (0.00–0.09)	665,886	804 (0.1%)	0.15 (0.07–0.22)
Leukaemia	857,320	3754 (0.4%)	0.70 (0.55–0.87)	380,799	1237 (0.3%)	0.59 (0.42–0.75)	476,521	2517 (0.5%)	0.80 (0.59–1.07)
Liver	1,287,960	4176 (0.3%)	0.63 (0.37–0.78)	610,967	1064 (0.2%)	0.37 (0.18–0.58)	676,993	3112 (0.5%)	0.79 (0.53–1.03)
Lung	1,298,771	13756 (1.1%)	1.62 (1.32–1.99)	614,990	4801 (0.8%)	1.35 (1.05–1.75)	683,781	8955 (1.3%)	1.81 (1.46–2.33)
Melanoma	1,277,044	11045 (0.9%)	0.80 (0.53–1.19)	603,754	4187 (0.7%)	0.54 (0.34–0.82)	673,290	6858 (1.0%)	1.00 (0.68–1.29)
Multiple myeloma	1,282,202	3246 (0.3%)	0.37 (0.22–0.56)	608,455	1218 (0.2%)	0.29 (0.17–0.49)	673,747	2028 (0.3%)	0.39 (0.24–0.70)
Ovary	603,034	2152 (0.4%)	0.50 (0.34–0.63)	603,034	2152 (0.4%)	0.50 (0.34–0.63)			
Pancreas	1,294,017	4272 (0.3%)	0.53 (0.38–0.62)	612,861	1574 (0.3%)	0.45 (0.30–0.57)	681,156	2698 (0.4%)	0.61 (0.39–0.71)
Prostate	682,001	22805 (3.3%)	4.91 (3.54–6.44)				682,001	22805 (3.3%)	4.91 (3.54–6.44)
Rectum	1,272,015	4193 (0.3%)	0.42 (0.31–0.48)	601,862	1548 (0.3%)	0.34 (0.30–0.45)	670,153	2645 (0.4%)	0.47 (0.30–0.55)
Stomach	1,277,051	2183 (0.2%)	0.26 (0.19–0.33)	603,756	602 (0.1%)	0.18 (0.10–0.23)	673,295	1581 (0.2%)	0.34 (0.25–0.43)
Thyroid	1,274,993	2046 (0.2%)	0.37 (0.24–0.47)	604,771	1286 (0.2%)	0.52 (0.19–0.73)	670,222	760 (0.1%)	0.20 (0.13–0.34)
Ureter	1,265,236	444 (0.0%)	0.06 (0.02–0.08)	599,350	120 (0.0%)	0.02 (0.00–0.06)	665,886	324 (0.0%)	0.07 (0.04–0.12)
Urological	1,265,438	12635 (1.0%)	1.66 (1.37–2.04)	599,260	2884 (0.5%)	0.96 (0.67–1.28)	666,178	9751 (1.5%)	2.29 (1.90–2.72)

^a^Participants who had both ACR and dipstick measures may have been included. We did not evaluate breast, ovarian or cervical cancer in men or prostate cancer in women. All cohorts did not contribute information on all subtypes of cancer. The Rancho Bernardo cohort did not contribute information on overall cancer.

### Overall cancer risk according to albuminuria and eGFR

**Fig. 1:**
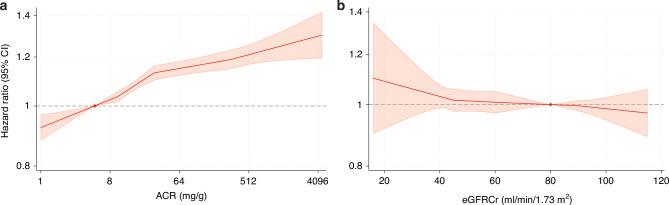
Adjusted hazard ratios of overall cancer using linear spline models in the ACR population. **a** Hazard ratios for overall cancer and ACR were adjusted for age, sex, body mass index, hypertension, diabetes, total cholesterol, use of aspirin or glucocorticoids, history of cardiovascular disease, current and former smoking, and eGFR. **b** Hazard ratios for overall cancer and eGFR were adjusted for age, sex, body mass index, hypertension, diabetes, total cholesterol, use of aspirin or glucocorticoids, history of cardiovascular disease, current and former smoking, and ACR.

In the categorical analysis of ACR, compared with the reference category of <10 mg/g, the higher categories were each associated with a higher risk of overall cancer (adjusted hazard ratio (HR), 1.10 [95% CI 1.07–1.12], 1.16 [1.12–1.20] and 1.26 [1.22–1.30] in 10–29 mg/g, 30–299 mg/g and ≥300 mg/g, respectively) (Table 3). In contrast, lower eGFRcr was not associated with the risk of overall cancer. The continuous analyses showed a robust dose-response relationship between ACR and cancer risk (Fig. 1). eGFRcr <45 mL/min/1.73 m^2^ appeared to reflect an increased risk of cancer compared to eGFR at 80 mL/min/1.73 m^2^, but confidence intervals crossed the null. The patterns of risk associations were generally similar across demographic and clinical subgroups (Fig. S1). Between cohort types, results were largely consistent for ACR but showed more variation for eGFRcr (Fig. S2).

**Table 3:** Adjusted hazard ratios of the association of eGFRcr and albuminuria with overall cancer risk in the ACR population.

eGFRcr, mL/min/1.73 m^2^	ACR, mg/g				Combined^a^
	<10 (*N* = 715,603)	10–29 (*N* = 326,886)	30–299 (*N* = 214,727)	≥300 (*N* = 60,580)	
**≥105 (*N*** = **278,638)**	0.91 (0.83–0.99)	1.07 (0.97–1.18)	1.08 (0.98–1.19)	1.30 (1.16–1.45)	0.93 (0.88–0.99)
**90–104 (*N*** = **431,923)**	REF	1.09 (1.05–1.13)	1.18 (1.13–1.24)	1.28 (1.17–1.39)	REF
**60–89 (*N*** = **456,786)**	0.99 (0.95–1.03)	1.06 (1.01–1.12)	1.14 (1.08–1.20)	1.20 (1.10–1.31)	0.98 (0.94–1.01)
**45–59 (*N*** = **85,790)**	1.07 (1.00–1.13)	1.07 (0.98–1.17)	1.18 (1.09–1.28)	1.32 (1.19–1.45)	1.01 (0.96–1.07)
**30–44 (*N*** = **41,856)**	1.05 (0.94–1.17)	1.06 (0.97–1.15)	1.17 (1.07–1.28)	1.31 (1.21–1.42)	0.97 (0.92–1.04)
**<30 (*N*** = **22,803)**	1.21 (1.03–1.41)	1.25 (1.08–1.44)	1.21 (1.11–1.33)	1.33 (1.20–1.49)	1.00 (0.91–1.10)
Combined^b^	REF	1.10 (1.07–1.12)	1.16 (1.12–1.20)	1.26 (1.22–1.30)	

*HR* adjusted for age, sex, body mass index, hypertension, diabetes, total cholesterol, use of aspirin or glucocorticoids, history of cardiovascular disease, current and former smoking.

^a^The marginal estimates for eGFR categories are adjusted for log-transformed ACR without an interaction term.

^b^The marginal estimates for ACR categories are adjusted for eGFRcr as a continuous variable with a knot at 60 ml/min/1.73 m^2^.

Higher dipstick proteinuria was associated with overall cancer risk in a dose-dependent manner. Even trace proteinuria demonstrated a significant increase in cancer risk (adjusted HR 1.11 [95% CI 1.07–1.15]) (Table S4). Similar to the results with eGFRcr, there was no significant association between eGFRcr-cys and overall cancer (Fig. S3). Associations for ACR and eGFRcr were consistent when beginning follow-up after 1 year from baseline (Fig. S4).

### Site-specific cancer risk according to albuminuria and eGFR

**Fig. 2:**
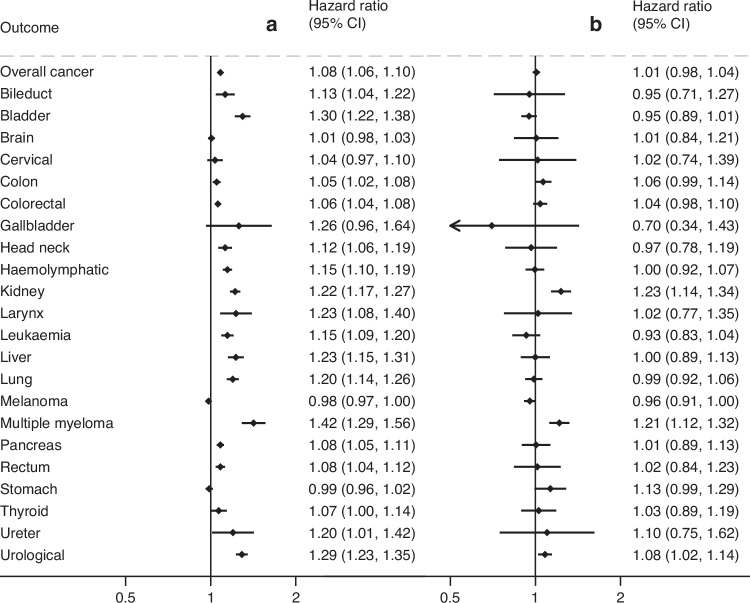
Adjusted hazard ratios of site-specific cancers in the ACR population. **a** Hazard ratios per 8-fold increase in ACR adjusted for age, sex, body mass index, hypertension, diabetes, total cholesterol, use of aspirin or glucocorticoids, history of cardiovascular disease, current and former smoking, and eGFR. **b** Hazard ratio per 15 ml/min/1.73 m^2^ decrease in eGFR creatinine in eGFR<60 ml/min/1.73 m^2^ adjusted for age, sex, body mass index, hypertension, diabetes, total cholesterol, use of aspirin or glucocorticoids, history of cardiovascular disease, current and former smoking, and ACR.

Higher ACR demonstrated significant associations with kidney, head and neck, colorectal, liver, pancreas, bile duct, larynx, lung, leukaemia, melanoma, ureter, urological, and hemolymphatic cancers, and multiple myeloma (Fig. 2a). Statistical significance was retained for all but bile duct, colon, melanoma, and ureter cancer after correction for multiple testing. In the dipstick proteinuria analysis, we observed similar associations (Fig. S5). Lower eGFRcr (per 15 mL/min/1.73m^2^ decrease) was associated with a higher risk of kidney cancer, urological cancer and multiple myeloma (Fig. 2). The results were generally consistent when beginning follow-up after 1 year from baseline (Fig. S4).

## Discussion

This international collaborative meta-analysis in >1.3 million individuals without cancer at baseline showed that higher ACR was independently associated with the risk of overall cancer, whereas the association was not evident with lower eGFR (neither eGFRcr nor eGFRcr-cys). We observed an increased risk even within the normal to mildly increased ACR (i.e., <30 mg/g), regardless of eGFR levels. The associations were largely consistent across demographic and clinical subgroups and when dipstick proteinuria was used as a measure of albuminuria. For site-specific cancers, lower eGFRcr showed a significant association with urological cancer and multiple myeloma, whereas higher ACR was related to broader types of cancer (e.g., head/neck, colorectal, liver, pancreas, bile duct, stomach, larynx, lung, hemolymphatic, leukaemia), in addition to kidney-related cancers.

Our study has some unique aspects, including a large sample size enabled through meta-analysis of individual-level data from international cohorts, simultaneous evaluation of different kidney measures, and assessment of the association with overall as well as site-specific cancers with detailed subgroup analyses. The incidence rate of overall cancer was around four times higher than in the general U.S. population (17.3 versus 4.4 per 1000 person-years) [23], likely reflecting selection of higher-risk populations with measurements of albuminuria. Despite these advantages, the association of cancer risk with eGFRcr was generally null, which is in line with several previous studies [5, 16]. Moreover, we found that even eGFRcr-cys, a more accurate estimate of GFR, was not associated with cancer risk. These results align well with a recent Mendelian Randomization study, which showed that genetically predicted eGFR was not associated with cancer risk [24]. Taken together, the aetiological contributions of reduced kidney function to overall cancer risk are likely limited.

In contrast, our study showed a strong association of higher ACR with cancer risk, which is in line with several previous epidemiological studies [5–7, 9, 10, 16, 18]. Though the mechanisms of association are uncertain, there are various potential explanations. First, individuals with albuminuria may serve as an indicator of increased susceptibility to cancer development. Beyond use as a marker of kidney damage, albuminuria has been implicated as both a cause and consequence of endothelial dysfunction and inflammation [25], and is seen more commonly among people with cardiometabolic disease, including diabetes, hypertension, and obesity. Further shared mechanisms exist that may exacerbate this relationship. For example, lifestyle factors, including a diet high in ultra-processed foods and reduced physical activity, are important risk factors that contribute to the development of metabolic diseases that can mutually exacerbate CKD progression and cancer risk [26]. Patients with CKD tend to have immunodeficiency [27], which can increase the risk of developing cancer [28]. The use of specific medications in the treatment of glomerular diseases (e.g., cyclophosphamide) can increase the risk of cancer, particularly bladder cancer [29]. Thus, albuminuria may serve as a marker of general ill health, with risk factors for and propensity to malignancy. Second, it is plausible that there are causal pathways between kidney dysfunction, including albuminuria, and cancer incidence. In one example, the kidney dysfunction and damage that predates kidney-related cancer incidence have been shown to be involved in cancer development pathways. Kidney damage and repair mechanisms that occur during acute kidney injury and CKD have been shown to trigger DNA damage and clonal proliferation of mutated cells, which was observed to drive tumorigenesis [30, 31]. Using Mendelian randomization studies, causal relationships have been demonstrated between albuminuria and cardiovascular disease (with a feed-forward mechanism mediated by blood pressure) [32] and mental disorders (through an yet unknown mechanism) [33]. Although a recent Mendelian Randomization study did not find an association between genetically-predicted albuminuria and cancer incidence, the genetic instrument explained small variation in ACR, and thus its adequacy in this context was uncertain [24]. Though further research is required to establish whether albuminuria is on the causal pathway, our robust dose-response results support the value of investigating the mechanisms behind the susceptibility of individuals with albuminuria to cancer development across a broad range of site-specific cancers.

When focusing on site-specific cancers, both kidney measures were consistently associated with the risk of urological cancer, and are in keeping with findings from previous studies [2–6, 8, 9, 14, 19]. In addition, our study showed a strong association of both eGFRcr and ACR with multiple myeloma, which is consistent with previous studies [5, 6]. The association seen between multiple myeloma, where immunoglobulin light chain in urine may worsen kidney function, suggests the possibility of reverse causality. However, when we began follow-up after 1 year from baseline, the association with baseline eGFR and multiple myeloma incidence remained significant.

Of note, lung cancer has consistently shown a robust association with albuminuria in studies, including ours [5, 6, 9, 10, 16]. The mechanisms regarding the relationship between albuminuria and lung cancer are not fully understood, and it is unclear what relevance albuminuria may have alongside other important risk markers for lung cancer, including smoking. For example, the incidence rate of lung cancer was around 3 times higher in the National Lung Screening Trial (a population of current or former heavy smokers ages 55 to 74) than in our ACR population [34]. However, there are multiple shared mechanisms that likely contribute. Inflammation plays a role in the development of cancer [35]. Vascular endothelial growth factor mediates angiogenesis, leads to albuminuria [36] and is overexpressed in patients with lung cancer [37]. Air pollutants and other toxic chemicals can cause both kidney disease and various cancers, including lung cancer [38]. Since our study is not designed to address mechanisms, further investigations are warranted to explore potential mechanisms behind the albuminuria-lung cancer relationship and whether albuminuria has any utility as a risk marker of cancer in selected populations.

Our study has clinical implications. Our findings suggest that CKD measures, particularly albuminuria, may be useful in identifying individuals at risk of cancer. Also, the robust association of albuminuria with lung cancer suggests exploration into whether the presence of albuminuria should indicate additional high-risk features that identify individuals at high risk of varied site-specific cancers. Since albuminuria assessment is recommended in several clinical conditions (e.g., diabetes, hypertension, and reduced eGFR) [39, 40], this measure is readily available in various clinical scenarios. However, additional studies would need to assess the effectiveness and cost-effectiveness of the use of albuminuria as a qualification metric for cancer screening, including the potential for unintended harm. In the meantime, healthcare providers should maintain a high index of suspicion in persons with albuminuria when presenting with symptoms or signs of cancer.

Although our study is the most comprehensive study to investigate the prospective association of CKD with cancer risk, we should acknowledge some important limitations. First, we describe associations between albuminuria, eGFR, and cancer incidence, but there remains substantial potential for measured and unmeasured confounding, as is true in all observational studies. Second, we do not have adequately granular data to present an analysis of the relationship between eGFR and albuminuria by cause of kidney disease. This may be of relevance where albuminuria and cancer are more likely to co-exist, such as in individuals with glomerulonephritis who are receiving immunosuppressive therapy. Third, the methods used to evaluate creatinine, albuminuria, and other covariates varied across cohorts despite our efforts to standardize definitions. However, this variation is unlikely to cause bias favouring kidney disease measures. Similarly, the ascertainment of cancer diagnoses was not necessarily consistent across cohort studies. Fourth, we do not have access to data concerning the genetic predisposition to cancer for individuals included in this study.

In conclusion, our results showed that albuminuria, but not necessarily eGFR (neither eGFRcr nor eGFRcr-cys), was robustly associated with the risk of overall cancer. Also, albuminuria was associated with a broad set of site-specific cancers, including a robust association with lung cancer. Our results warrant consideration of the mechanisms that explain the link between albuminuria and cancer risk across a broad range of site-specific cancers and the possible utility of albuminuria as a biomarker of future cancer risk.

## Supplementary information

Supplemental Appendices

Supplemental Tables and Figures excel file

## Data Availability

Under agreement with the participating cohorts, CKD-PC cannot share individual data with third parties. Inquiries regarding specific analyses should be made to ckdpc@nyulangone.org. Investigators may approach the original cohorts regarding their own policies for data sharing (e.g., https://aric.cscc.unc.edu/aric9/researchers/Obtain_Submit_Data for the Atherosclerosis Risk in Communities Study).
