# Supplementary material for: Chronic kidney disease and incident cancer risk: an individual participant data meta-analysis
Source: Br J Cancer. 2025 Sep 6;133(10):1535–43. doi: 10.1038/s41416-025-03140-z (PMC12603274; doi:10.1038/s41416-025-03140-z)
Supplement: Supplementary file 1 — Supplemental Appendices [file 41416_2025_3140_MOESM1_ESM.docx]

Supplementary Appendices

**Table of Contents**

[Appendix 1. Data analysis overview and analytic notes for some of individual studies 2](#_Toc187994825)

[Appendix 2. Acronyms or abbreviations for studies included in the current report and their key references linked to the Web references 8](#_Toc187994826)

[Appendix 3. Acknowledgements and funding for collaborating cohorts 9](#_Toc187994827)

[References 12](#_Toc187994828)

# Appendix 1. Data analysis overview and analytic notes for some of individual studies

- 1. Overview

To determine the cohorts eligible for this paper, in August/September 2022 all cohorts in CKD-PC were contacted regarding their interest in this topic and in May 2024 cohorts were contacted to confirm availability of cancer outcome data as well as required covariates. Of the 118 cohorts (31 OLDW cohorts) that were contacted, 53 cohorts opted in and had available data, 54 did not have the outcome or covariate data needed, 11 were not able to provide data or run code within our time frame to be included. Cohorts were required to have non-fatal cancer outcome event data and covariate information on BMI (at least 50% non-missing) and smoking (at least 80% non-missing).

As previously described,^1^ the collaborating cohorts were asked to prepare a dataset with approximately 30 variables (key exposures [serum creatinine to estimate GFR and albuminuria]), covariates [e.g., age, sex, race/ethnicity, diabetes, hypertension, body mass index, smoking status]), and outcomes [follow-up time and event variables]).

To minimize heterogeneity, the CKD-PC Data Coordinating Center (DCC) sent definitions of variables and dataset preparation (outlined below). We instructed studies not to impute any variables.

| **Medical history variable** | **Definition** |
| --- | --- |
| Smoking | Information on current-, former-, and never-smoker |
| Height | Height (m) |
| Body weight | Body weight (kg) |
| Diabetes mellitus | Glycated hemoglobin A1c ≥6.5% or fasting glucose ≥7.0 mmol/L (≥126 mg/dL) or non-fasting glucose ≥11.1 mmol/L (≥200 mg/dL) or use of glucose lowering drugs (ADA 2010 criteria). Self-report of physician diagnosed diabetes can be included.  Identification at any time during study period (from baseline to follow-up). |
| Systolic blood pressure | Systolic blood pressure (mmHg) |
| Hypertension | Systolic blood pressure ≥140 mmHg or diastolic blood pressure ≥90 mmHg or antihypertensive drugs (JNC-7 criteria).  Self-reported hypertension if other data not available.  Identification at any time during study period (from baseline to follow-up). |
| Medications for hypertension | Taking any medications prescribed for lowering blood pressure. In administrative data we used ATC codes: C02, C03, C07, C08, C09 |
| Urine protein-to-creatinine ratio (PCR) | Urinary protein (mg) / Urinary creatinine (g) |
| Dipstick proteinuria | Dipstick proteinuria |
| Serum creatinine | Serum creatinine standardized to isotope dilution mass spectrometry (IDMS) (mg/dL) -- specify assay and standardization method |
| Serum cystatin C | Serum cystatin C standardized to International Federation for Clinical Chemists (IFCC) (mg/dL) -- specify assay and standardization method |

Outcome definitions below were provided to cohorts, cohort deviations from these definitions listed in cohort specific notes.

| **Outcome** | **Definition** |
| --- | --- |
| Cancer | First incidence of cancer |

ICD codes used to define outcomes within cohorts, if not specified in cohort specific notes

| **Cancer** | **ICD-9 codes** | **ICD-10 codes** |
| --- | --- | --- |
| Any | 140-210 (except for 173) | C00-C99 (except for C44) |
| Bile Duct | 156.1, 156.2, 156.8, 156.9 | C24 |
| Bladder | 188 | C67 |
| Brain | 191-192 | C70-C72 |
| Breast (only women) | 174-175 | C50 |
| Cervix (only women) | 180 | C53 |
| Colon | 153 | C18 |
| Gallbladder | 156.0 | C23 |
| Head and Neck | 140-149 | C00-C14, C30-C32 |
| Hematologic Malignancies | 200-208 | C81-C86, C88 |
| Kidney | 189.0 | C64 |
| Larynx | 161 | C32 |
| Liver | 155 | C22 |
| Lung | 162 | C33-C34 |
| Melanoma | 172 | C43 |
| Multiple Myeloma | 203.0 | C90.0 |
| Ovary (only women) | 183.0 | C56 |
| Pancreas | 157 | C25 |
| Prostate | 185 | C61 |
| Rectum | 154 | C19-C20 |
| Stomach | 151 | C16 |
| Thyroid | 193 | C73 |
| Ureter | 189.2 | C66 |

The CKD-PC data request and processing procedures are as follows. After obtaining opt-in preferences from cohorts for the topics for each phase, the Data Coordinating Center (DCC) requests de-identified data using a specific data request document describing the variables and preferred definitions needed for the current phase of the CKD-PC. Cohorts work with the DCC on any data use agreements, IRB approvals, and other logistic issues for de-identified data transfer. The DCC also advises on any differences in definitions or questions on data formatting. Cohorts then provide de-identified data (in whatever program format, e.g., Stata, SAS, csv) via a secure data transfer provided or their own secure transfer program/platform or they created the dataset locally to then run standardized code. Data or outputs are stored on a secure password protected network server that is accessed only by limited faculty and staff (<10). All those faculty and staff have completed HIPAA and CITI certification and have signed internal data use agreements to not use the data for any other than stated purposed and to not remove the data from that network drive. The CKD-PC does not share data with any external parties. Once data is received and stored in the network drive, the DCC programmer reviews the data and the data dictionary provided by the cohort to check for any missing information, outliers, and potentials issues with variable units, dates, etc. Any questions are sent to the cohort representatives for data checking and cleaning. Further data checking is done throughout the analysis process for each CKD-PC paper, including a review from a cohort representative of all tables and figures to confirm their cohort representation.

For 45 of 54 cohorts in this specific study, the DCC at the New York University Grossman School of Medicine conducted the analysis; the remainder ran the standard code written in Stata by the DCC and shared the output with the DCC (BioVU, CARE, CKD-REIN, Go-DARTs, KCPS-II, RCAV, SEED, TaiwanMJ, UKBiobank). Standard code was designed to automatically save all estimates and variance-covariance matrices needed for the meta-analysis. Then the DCC meta-analyzed the estimates across cohorts using Stata.

As detailed in our previous reports,^2,3^ each cohort was instructed to standardize their serum creatinine and report its method when available. The reported creatinine standardization allows grouping studies into studies that reported using a standard IDMS traceable method or conducted some serum creatinine standardization to IDMS traceable methods (ARIC, BioVU, CARE, CKD-REIN, CRIC, ELSA-Brasil, ESTHER, GCKD, Geisinger, Go-DARTs, MESA, NYU, PREVEND, Rancho Bernardo, RCAV, SEED, UK Biobank) and studies where the creatinine standardization was not done (ADVANCE, KCPS-II, Taiwan MJ, ULSAM). For those cohorts without standardization, the creatinine levels were reduced by 5%, the calibration factor used to adjust non-standardized MDRD Study samples to IDMS.^2,4^ We did not adjust creatinine levels in those studies with unknown standardization status(SHARP, OLDW all cohorts, SKS).

Serum cystatin C values were calibrated and/or standardized to International Federation for Clinical Chemists (IFCC).^5,6^ Cohort details below:

| Cohort | Notes including if a calibration equation was applied |
| --- | --- |
| ARIC | IFCC Cystatin C = 1.12*(0.083+0.914*(ARIC cystatin C)) |
| CKD_REIN | ERM-DA471/IFCC Standardized assay |
| CRIC | Calibrated by primary study |
| ESTHER | IFCC= 1.12*(0.105+0.848*(ESTHER cystatin C)) |
| GCKD | ERM-DA471/IFCC Standardized assay |
| MESA | IFCC= 1.12*(0.083+0.789*(MESA cystatin C)) |
| PREVEND | IFCC= 1.12*(0.083+0.789*(MESA cystatin C)) |
| UKBioBank | ERM-DA471/IFCC Standardized assay |
| ULSAM | IFCC= 1.12*(0.083+0.789*(ULSAM cystatin C)) |

We calculated creatinine and creatinine-cystatin C based eGFR using the 2021 CKD-EPI equation^7^ and cystatin C based eGFR using the 2012 CKD-EPI equation,^8^ as follows:

| Sex | Serum Creatinine (mg/dL) | Serum Cystatin C (mg/dL) | Equation |
| --- | --- | --- | --- |
| CKD-EPI creatinine equation (2021) | | | |
| Female | ≤0.7 |  | GFR= 142 x (Scr/0.7)^-0.241^ x 0.9938^Age^ |
|  | >0.7 |  | GFR= 142 x (Scr/0.7)^-1.200^ x 0.9938^Age^ |
| Male | ≤0.9 |  | GFR= 142 x (Scr/0.9)^-0.302^ x 0.9938^Age^ |
|  | >0.9 |  | GFR= 142 x (Scr/0.9)^-1.200^ x 0.9938^Age^ |
| CKD-EPI creatinine-cystatin C equation (2021) | | | |
| Female | ≤0.7 | ≤0.8 | GFR= 135 x (Scr/0.7)^-0.219^ x (Scys/0.8)^-0.323^ x 0.9961^Age^ x 0.963 |
|  |  | >0.8 | GFR= 135 x (Scr/0.7)^-0.544^ x (Scys/0.8)^-0.778^ x 0.9961^Age^ x 0.963 |
|  | >0.7 | ≤0.8 | GFR= 135 x (Scr/0.7)^-0.544^ x (Scys/0.8)^-0.778^ x 0.9961^Age^ x 0.963 |
|  |  | >0.8 | GFR= 135 x (Scr/0.7)^-0.544^ x (Scys/0.8)^-0.778^ x 0.9961^Age^ x 0.963 |
| Male | ≤0.9 | ≤0.8 | GFR= 135 x (Scr/0.9)^-0.144^ x (Scys/0.8)^- 0.323^ x 0.9961^Age^ |
|  |  | >0.8 | GFR= 135 x (Scr/0.9)^-0.144^ x (Scys/0.8)^- 0.323^ x 0.9961^Age^ |
|  | >0.9 | ≤0.8 | GFR= 135 x (Scr/0.9)^-0.144^ x (Scys/0.8)^- 0.323^ x 0.9961^Age^ |
|  |  | >0.8 | GFR= 135 x (Scr/0.9)^-0.544^ x (Scys/0.8)^- 0.778^ x 0.9961^Age^ |

The selection of knots for eGFR and urine albumin-to-creatinine ratio (ACR) was based on clinical thresholds.^9^ Baseline for each study was considered first available creatinine unless otherwise noted. Other variables were taken either on baseline date or within one year before baseline date.

By definition, age, sex, BMI, ACR, and eGFR were never missing. Within each cohort, if smoking was missing, participants were dropped in research cohort or imputed as never smoker in Electronic Health Record (EHR).

- 1. Notes for individual cohorts

| Cohort | Study Design | Baseline Year(s) | Albuminuria type(s) | Cystatin C available | Specific notes |
| --- | --- | --- | --- | --- | --- |
| ADVANCE | Clinical trial cohort | 2001-03 | ACR | No | This study is a clinical trial which includes participants with diabetes only. All outcomes were actively ascertained and verified by an adjudication committee blinded to the randomized treatment |
| ARIC | Research cohort | 1996-98 | ACR | Yes | Visit 4 was used as the baseline. All-cause mortality was actively ascertained as well as through linkage to a registry. Cancer incidences were ascertained by linkage to state cancer registries of the states of North Carolina, Mississippi, Minnesota, and Maryland, and supplemented by abstraction of medical records and hospital discharge codes for self-reported cases. Participants who self-reported a diagnosis of cancer on an annual follow-up telephone call (semiannual from 2012) were contacted separately for more information on cancer diagnoses, and medical records pertaining to cancer diagnoses and treatment were collected. High priority cancers (bladder, breast, colorectal, liver, lung, pancreas, and prostate) not captured by registries were confirmed by an adjudication team through all collected materials. Other types of cancer were confirmed through the cancer registry, abstraction of archived medical records for time intervals not covered by the registry, and death certificate data, but they were not otherwise adjudicated. |
| BioVU | Clinical database | 2016-2024 | ACR, PCR, Dipstick | No | Baseline index date was set as the earliest date of a serum creatinine measurement after 2016 and at least one year after enrollment. All outcomes were defined by ICD codes from encounters. |
| CARE | Clinical trial cohort | 1989-90 | Dipstick | No | All outcomes were actively ascertained by an independent review committee. |
| CKD-REIN | Research cohort | 2013-16 | ACR, PCR | Yes | All outcomes were actively ascertained. All-cause mortality and the initiation of kidney replacement therapy were further ascertained through registry linkage. |
| CRIC | Research cohort | 2003-2008 | ACR | Yes | All-cause mortality was actively ascertained. |
| ELSA-Brasil | Research cohort | 2008-2010 | ACR | No | All-cause mortality was actively ascertained from medical records and linkage to the official health statistics databank. Cancer surveillance and investigation involved annual telephone interviews, data collection of medical registries (biopsy records, chemotherapy/radiotherapy reports, hospital records or death certificates/autopsy statements), eligibility and final classification, date and basis of diagnosis, tumor topography and histological type. |
| ESTHER | Research cohort | 2000-02 | Dipstick | Yes | All outcomes were actively ascertained by questionnaires sent to the study participants and their general practitioners 2, 5, 8, 11 and 14 years after the cohort’s baseline assessment. In addition, cancer cases were ascertained by record linkage with the population-based Saarland Cancer Registry, and fatal disease events were ascertained by a mortality register and ICD-10 codes of the leading cause of death on the death certificate. |
| GCKD | Research Cohort | 2010-12 | ACR | Yes | All outcomes were actively ascertained (with confirmation in medical chart review).^10^ |
| Geisinger | Healthcare administrative database | 2008-19 | ACR, PCR, Dipstick | No | Baseline index date was set as the earliest date of a serum creatinine measurement after 2008 and at least one year after enrollment. |
| Go-DARTs | Research cohort | 2004-12 | ACR | No | Baseline index date was set as the earliest date of a serum creatinine measurement. |
| KCPS-II | Research Cohort | 2004-13 | Dipstick | No | All-cause mortality was ascertained by linkage to a death registry. Cancer was ascertained by linkage to the national cancer center registry. |
| MESA | Research cohort | 2000-02 | ACR | Yes | All participants free from previous cardiovascular disease at baseline. All-cause mortality was actively ascertained. |
| NYU | Healthcare administrative database | 2010-2024 | ACR/PCR | No | Baseline index date was set as the earliest date of a serum creatinine measurement at least one year after enrollment. |
| OLDW 1-31 | Healthcare administrative database | 2012-2021 | ACR, PCR, Dipstick | No | This study used de-identified electronic health record (EHR) data from the Optum Labs Data Warehouse (OLDW). The database contains longitudinal health information on enrollees and patients, representing a diverse mixture of ages and geographical regions across the United States. The EHR-derived data includes a subset of EHR data that has been normalized and standardized into a single database.^11^ Cohort inclusion criteria was more than 50 events of any outcome before excluding missing values of main exposure variables. Smoking status might be under measured in this study. All outcomes were defined by ICD codes from encounters. No cause of death information available. Baseline index date was set as the earliest date of a serum creatinine measurement after 2008 and at least one year after enrollment. |
| PREVEND | Research cohort | 1997-98 | ACR | Yes | All-cause mortality was ascertained by linkage to a death registry. |
| Rancho Bernardo | Research cohort | 1992-96 | ACR | No | All-cause mortality was ascertained by linkage to a death registry. |
| RCAV | Healthcare administrative database | 2004-2011 | ACR, PCR | No | All outcomes were defined by ICD codes from encounters. Baseline index date was set as the earliest date of a serum creatinine at least one year after enrollment. |
| SEED | Research cohort | 2004-11 | ACR | No | All-cause mortality and cancer were ascertained by linkage to National Disease Registry and Death Registry. |
| SHARP | Clinical trial cohort | 2003-2007 | ACR | No | All outcomes were actively ascertained. |
| SKS | Research cohort | 2002-16 | PCR | No | All outcomes were actively ascertained (with physician panel adjudication). |
| Taiwan MJ | Research cohort | 1994-2011 | Dipstick | No | Cancer was ascertained from ICD codes. |
| UK BioBank | Clinical database | 2007-10 | ACR | Yes | All-cause mortality was ascertained by linkage to a death registry. Cancer events were ascertained by ICD codes at hospital discharge. |
| ULSAM | Research cohort | 1991-95 | ACR | Yes | All-cause mortality was ascertained by linkage to a death registry. Cancer events were ascertained by ICD codes at hospital discharge. |

*Type of albuminuria used in analyses with a preference for ACR. Does not necessarily indicate all types available within the cohort.

# Appendix 2. Acronyms or abbreviations for studies included in the current report and their key references linked to the Web references

| ADVANCE | The Action in Diabetes and Vascular Disease: Preterax and Diamicron Modified Release Controlled Evaluation (ADVANCE) trial^12^ |
| --- | --- |
| ARIC | Atherosclerosis Risk in Communities Study^13^ |
| BioVU | BioVU: Vanderbilt University Medical Center’s De-Identified Biobanking Program^14^ |
| CARE | The Cholesterol and Recurrent Events (CARE) Trial^15^ |
| CKD-REIN | Chronic Kidney Disease - Renal Epidemiology and Information Network (CKD-REIN) cohort study^16^ |
| CRIC | Chronic Renal Insufficiency Cohort Study^17^ |
| ELSA-Brasil | Longitudinal Study of Adult Health (BRAZIL)^18,19^ |
| ESTHER | Epidemiologische Studie zu Chancen der Verhütung, Früherkennung und optimierten THerapie chronischer ERkrankungen in der älteren Bevölkerung [GERMAN]^20^ |
| GCKD | German Chronic Kidney Disease study^21^ |
| Geisinger | Geisinger Health System^22^ |
| Go-DARTs | Genetics of Diabetes Audit and Research in Tayside Scotland^23^ |
| KCPS-II | Korean Cancer Prevention Study-II^24^ |
| MESA | Multi-Ethnic Study of Atherosclerosis^25^ |
| NYU | New York University Medical System |
| OLDW | Optum Labs Data Warehouse |
| PREVEND | Prevention of Renal and Vascular End-stage Disease Study^26^ |
| Rancho Bernardo | Rancho Bernardo Study^27^ |
| RCAV | Racial and Cardiovascular Risk Anomalies in CKD Cohort^28^ |
| SEED | Singapore Epidemiology of Eye Diseases^29^ |
| SHARP | Study of Heart and Renal Protection |
| SKS | Salford Kidney Study^30^ |
| TaiwanMJ | Taiwan MJ Cohort Study^31^ |
| UK BioBank | The United Kingdom Biobank Study^32^ |
| ULSAM | Uppsala Longitudinal Study of Adult Men^33^ |

# Appendix 3. Acknowledgements and funding for collaborating cohorts

| **Cohort** | **List of sponsors** |
| --- | --- |
| ADVANCE | ADVANCE was supported by research grants from Servier International and from the National Health and Medical Research Council (NHMRC) of Australia program grants 358395, 571281, 1052555 and 1149987 and project grant 211086 |
| ARIC | The Atherosclerosis Risk in Communities study has been funded in whole or in part with Federal funds from the National Heart, Lung, and Blood Institute, National Institutes of Health, Department of Health and Human Services, under Contract nos. (75N92022D00001, 75N92022D00002, 75N92022D00003, 75N92022D00004, 75N92022D00005). Studies on cancer in ARIC are also supported by the National Cancer Institute (U01 CA164975). The content of this work is solely the responsibility of the authors and does not necessarily represent the official views of the National Institutes of Health.  Cancer data was provided by the Maryland Cancer Registry, Center for Cancer Prevention and Control, Maryland Department of Health, with funding from the State of Maryland and the Maryland Cigarette Restitution Fund. The collection and availability of cancer registry data are also supported by the Cooperative Agreement NU58DP007114, funded by the Centers for Disease Control and Prevention. Its contents are solely the responsibility of the authors and do not necessarily represent the official views of the Centers for Disease Control and Prevention or the Department of Health and Human Services.  The authors thank the staff and participants of the ARIC study for their important contributions |
| BioVU | The BioVU projects at Vanderbilt University Medical Center are supported by numerous  sources: institutional funding, private agencies, and federal grants. These include the NIH-funded Shared Instrumentation Grant S10OD017985 and S10RR025141; CTSA grants  UL1TR002243, UL1TR000445, and UL1RR024975 from the National Center for Advancing Translational Sciences. Its contents are solely the responsibility of the authors and do not necessarily represent official views of the National Center for Advancing Translational Sciences or the National Institutes of Health. |
| CARE | Foundation grant from Canadian Institutes of Health Research to Dr. Tonelli (FRN 143211) |
| CKD-REIN | CKD-REIN is funded by the Agence Nationale de la Recherche through the 2010 «Cohortes-Investissements d’Avenir » program (ANR-IA-COH-2012/3731) and by the 2010 national Programme Hospitalier de Recherche Clinique. CKD-REIN is also supported through a public-private partnership GlaxoSmithKline (GSK) since 2012, Boehringer Ingelheim France since 2022, Fresenius Medical Care from 2012 to 2024, Vifor France from 2018 to 2023, Sanofi-Genzyme from 2012 to 2015, Baxter and Merck Sharp & Dohme-Chibret (MSD France) from 2012 to 2017, Amgen from 2012 to 2020, Lilly France from 2013 to 2018, Otsuka Pharmaceutical from 2015 to 2020, and AstraZeneca from 2018 to 2021. |
| CRIC | Funding for the CRIC Study was obtained under a cooperative agreement from National Institute of Diabetes and Digestive and Kidney Diseases (*U01DK060990, U01DK060984, U01DK061022, U01DK061021, U01DK061028, U01DK060980, U01DK060963, U01DK060902 and U24DK060990*). In addition, this work was supported in part by: the Perelman School of Medicine at the University of Pennsylvania Clinical and Translational Science Award NIH/NCATS *UL1TR000003*, Johns Hopkins University *UL1 TR-000424*, University of Maryland *GCRC M01 RR-16500*, Clinical and Translational Science Collaborative of Cleveland, *UL1TR000439* from the National Center for Advancing Translational Sciences (NCATS) component of the National Institutes of Health and NIH roadmap for Medical Research, Michigan Institute for Clinical and Health Research (MICHR) *UL1TR000433*, University of Illinois at Chicago CTSA *UL1RR029879*, Tulane COBRE for Clinical and Translational Research in Cardiometabolic Diseases *P20 GM109036*, Kaiser Permanente NIH/NCRR *UCSF-CTSI UL1 RR-024131*, Department of Internal Medicine, University of New Mexico School of Medicine Albuquerque, *NM R01DK119199*. |
| ELSA-Brasil | The ELSA-Brasil baseline study is supported by the Brazilian Ministry of Health (Science, Technology and Innovation Department) and the Brazilian Ministry of Science and Technology (FINEP - Financiadora de Estudos e Projetos, and CNPq - National Research Council) (Baseline grants 0106 0010.00 RS, 01 06 0212.00 BA, 01 060300.00 ES, 01 06 0278.00 MG, 01 060115.00 SP, 01 06 0071.00 RJ). SMB, PAL and JGM are research fellows from CNPq (Brazilian National Research Council, grants |
| ESTHER | Ministry of Research, Science and the Arts Baden-Württemberg (Stuttgart, Germany), Federal Ministry of Education and Research (Berlin, Germany), Federal Ministry of Family Affairs, Senior Citizens, Women and Youth (Berlin, Germany), Saarland state Ministry for Social Affairs, Health, Women and Family Affairs (Saarbrücken, Germany). Measurement of urinary albumin was funded by Dade-Behring, Marburg, Germany. |
| GCKD | The GCKD study is supported by grants from the Federal Ministry of Education and Research (Bundesministerium für Bildung und Forschung; www.bmbf.de), FKZ 01ER 0804, 01ER 0818, 01ER 0819, 01ER 0820, 01ER 0821, and 01ER 0822, and the Foundation for Preventive Medicine of the KfH (Kuratorium für Heimdialyse und Nierentransplantation e.V. – Stiftung Präventivmedizin; [www.kfh-stiftung-praeventivmedizin.de](http://www.kfh-stiftung-praeventivmedizin.de)) and corporate partners (for a list see [www.gckd.org](http://www.gckd.org)). The GCKD investigators gratefully acknowledge the expert support of all members of study staff, the dedicated contribution of all collaborating nephrologists (for a list of contributors and the 169 study sites, see www.gckd.org) and the support of patients participating in the study. The work of AK was supported by the Deutsche Forschungsgemeinschaft (DFG, German Research Foundation) Project ID 431984000 SFB 1453. The work of UTS was supported by the German Federal Ministry of Education and Research (BMBF) within the framework of the e:Med research and funding concept (grant 01ZX1912B). |
| Geisinger | Geisinger Clinic; NIDDK R01DK100446 |
| GoDARTS | The Wellcome Trust United Kingdom Type 2 Diabetes Case Control Collection (supporting GoDARTS) was funded by the Wellcome Trust, under grants 072960/Z/03/Z, 084726/Z/08/Z, 084727/Z/08/Z, 085475/Z/08/Z, and 085475/B/08/Z. |
| KCPS-II | This study was supported by grant from the Basic Science Research Program through the National Research Foundation of Korea funded by the Ministry of Education (RS-2023-00239122) |
| MESA | This research was supported by contracts HHSN268201500003I, N01-HC-95159, N01-HC-95160, N01-HC-95161, N01-HC-95162, N01-HC-95163, N01-HC-95164, N01-HC-95165, N01-HC-95166, N01-HC-95167, N01-HC-95168 and N01-HC-95169 from the National Heart, Lung, and Blood Institute and by grants UL1-TR-000040 and UL1-TR-001079 from NCRR. The authors thank the other investigators, the staff, and the participants of the MESA study for their valuable contributions. A full list of participating MESA investigators and institutions can be found at <http://www.mesa-nhlbi.org>. |
| NYU |  |
| OLDW | N/A |
| PREVEND | The PREVEND study is supported by several grants from the Dutch Kidney Foundation, and grants from the Dutch Heart Foundation, the Dutch Government (NWO), the US National Institutes of Health (NIH) and the University Medical Center Groningen, The Netherlands (UMCG). Dade Behring, Marburg, Germany supplied equipment and reagents for nephelometric measurement of urinary albumin. |
| Rancho Bernardo | NIA AG07181 and AG028507 NIDDK DK31801 |
| RCAV | This study was supported by grant R01DK096920 from NIH-NIDDK and is the result of work supported with resources and the use of facilities at the Memphis VA Medical Center and the Long Beach VA Medical Center. Support for VA/CMS data is provided by the Department of Veterans Affairs, Veterans Health Administration, Office of Research and Development, Health Services Research and Development, VA Information Resource Center (project numbers SDR 02-237 and 98-004). |
| SEED | This study was supported by grants from the Singapore Ministry of Health's National Medical Research Council (NMRC), NMRC/STaR/0003/2008, NMRC/0796/2003, NMRC/1249/2010, NMRC/TA/0008/2012, Duke-NUS-KMRA/2015/0003, NMRC CIRG/1371/2013, NMRC/STaR/016/2013/ and NMRC/OFLCG/001/2017. |
| SHARP | SHARP was funded by Merck/Schering-Plough Pharmaceuticals (North Wales, PA), with additional support from the Australian National Health and Medical Research Council, the British Heart Foundation, and the UK Medical Research Council. SHARP was initiated, conducted, and interpreted independently of the principal study funder (Merck & Co. and Schering Plough Corp., which merged in 2009). The authors thank the participants in the SHARP trials, as well as the  local clinical center staff, regional and national coordinators, steering committees, and data monitoring committees. |
| SKS | Support received from the local LCRN and funded by Investigator-Initiated (IIT) grants from Vifor, Astellas, Bergen Bio and EVOTEC |
| TaiwanMJ | This study was supported in part by Taiwan Ministry of Health and Welfare Clinical Trial Center (MOHW109-TDU-B-212-114004), MOST Clinical Trial Consortium for Stroke (MOST 109-2321-B-039-002), China Medical University Hospital (DMR-109-231), Tseng-Lien Lin Foundation, Taichung, Taiwan. |
| UKBioBank | The UK Biobank was supported by the Medical Research Council, the Wellcome Trust, the UK Department of Health, the British Heart Foundation, Cancer Research UK, the US National Institute for Health Research, the Scottish Government, the North West Development Agency, Diabetes UK, and the Welsh Government (grants are listed here https://www.ukbiobank.ac.uk/wp-content/uploads/2018/10/Funding-UK-Biobank-summary.pdf). |
| ULSAM | The Swedish Research Council, the Swedish Heart-Lung Foundation, the Marianne and Marcus Wallenberg Foundation, Dalarna University, and Uppsala University. |

# References

**1.** Chronic Kidney Disease Prognosis Consortium, Matsushita K, van der Velde M, et al. Association of estimated glomerular filtration rate and albuminuria with all-cause and cardiovascular mortality in general population cohorts: a collaborative meta-analysis. *Lancet.* Jun 12 2010;375(9731):2073-2081.

**2.** Matsushita K, Mahmoodi BK, Woodward M, et al. Comparison of risk prediction using the CKD-EPI equation and the MDRD study equation for estimated glomerular filtration rate. *JAMA.* May 9 2012;307(18):1941-1951.

**3.** Hallan SI, Matsushita K, Sang Y, et al. Age and association of kidney measures with mortality and end-stage renal disease. *JAMA.* Dec 12 2012;308(22):2349-2360.

**4.** Levey AS, Coresh J, Greene T, et al. Expressing the Modification of Diet in Renal Disease Study equation for estimating glomerular filtration rate with standardized serum creatinine values. *Clin Chem.* Apr 2007;53(4):766-772.

**5.** Grubb A, Blirup-Jensen S, Lindstrom V, et al. First certified reference material for cystatin C in human serum ERM-DA471/IFCC. *Clin Chem Lab Med.* Nov 2010;48(11):1619-1621.

**6.** Inker LA, Eckfeldt J, Levey AS, et al. Expressing the CKD-EPI (Chronic Kidney Disease Epidemiology Collaboration) cystatin C equations for estimating GFR with standardized serum cystatin C values. *Am J Kidney Dis.* Oct 2011;58(4):682-684.

**7.** Inker LA, Eneanya ND, Coresh J, et al. New Creatinine- and Cystatin C-Based Equations to Estimate GFR without Race. *N Engl J Med.* Nov 4 2021;385(19):1737-1749.

**8.** Inker LA, Schmid CH, Tighiouart H, et al. Estimating glomerular filtration rate from serum creatinine and cystatin C. *N Engl J Med.* Jul 5 2012;367(1):20-29.

**9.** Kidney Disease: Improving Global Outcomes C. K. D. M. B. D. Update Work Group. KDIGO 2017 Clinical Practice Guideline Update for the Diagnosis, Evaluation, Prevention, and Treatment of Chronic Kidney Disease-Mineral and Bone Disorder (CKD-MBD). *Kidney Int Suppl (2011).* Jul 2017;7(1):1-59.

**10.** Steinbrenner I, Kotsis F, Kosch R, et al. Interactive exploration of adverse events and multimorbidity in CKD. *Nephrol Dial Transplant.* Apr 25 2024.

**11.** Optum Labs. *Optum Labs and OptumLabs Data Warehouse (OLDW) Descriptions and Citation.* Eden Prairie, MN: n.p.;March 2023.

**12.** Patel A, MacMahon S, Chalmers J, et al. Effects of a fixed combination of perindopril and indapamide on macrovascular and microvascular outcomes in patients with type 2 diabetes mellitus (the ADVANCE trial): a randomised controlled trial. *Lancet.* Sep 8 2007;370(9590):829-840.

**13.** Matsushita K, Selvin E, Bash LD, Franceschini N, Astor BC, Coresh J. Change in estimated GFR associates with coronary heart disease and mortality. *J Am Soc Nephrol.* Dec 2009;20(12):2617-2624.

**14.** Ritchie MD, Denny JC, Crawford DC, et al. Robust replication of genotype-phenotype associations across multiple diseases in an electronic medical record. *Am J Hum Genet.* Apr 9 2010;86(4):560-572.

**15.** Tonelli M, Jose P, Curhan G, et al. Proteinuria, impaired kidney function, and adverse outcomes in people with coronary disease: analysis of a previously conducted randomised trial. *BMJ.* Jun 17 2006;332(7555):1426.

**16.** Stengel B, Metzger M, Combe C, et al. Risk profile, quality of life and care of patients with moderate and advanced CKD: The French CKD-REIN Cohort Study. *Nephrol Dial Transplant.* Feb 1 2019;34(2):277-286.

**17.** Denker M, Boyle S, Anderson AH, et al. Chronic Renal Insufficiency Cohort Study (CRIC): Overview and Summary of Selected Findings. *Clin J Am Soc Nephrol.* Nov 6 2015;10(11):2073-2083.

**18.** Schmidt MI, Duncan BB, Mill JG, et al. Cohort Profile: Longitudinal Study of Adult Health (ELSA-Brasil). *Int J Epidemiol.* Feb 2015;44(1):68-75.

**19.** Moreira AD, Teles de Menezes S, Junior MM, da Silva Vicente JT, Camelo LV, Barreto SM. Investigation of cancer incidence in ELSA-Brasil. *Cancer Epidemiol.* Dec 2023;87:102467.

**20.** Zhang QL, Koenig W, Raum E, Stegmaier C, Brenner H, Rothenbacher D. Epidemiology of chronic kidney disease: results from a population of older adults in Germany. *Prev Med.* Feb 2009;48(2):122-127.

**21.** Titze S, Schmid M, Kottgen A, et al. Disease burden and risk profile in referred patients with moderate chronic kidney disease: composition of the German Chronic Kidney Disease (GCKD) cohort. *Nephrol Dial Transplant.* Mar 2015;30(3):441-451.

**22.** Perkins RM, Bucaloiu ID, Kirchner HL, Ashouian N, Hartle JE, Yahya T. GFR decline and mortality risk among patients with chronic kidney disease. *Clin J Am Soc Nephrol.* Aug 2011;6(8):1879-1886.

**23.** Hebert HL, Shepherd B, Milburn K, et al. Cohort Profile: Genetics of Diabetes Audit and Research in Tayside Scotland (GoDARTS). *Int J Epidemiol.* Apr 1 2018;47(2):380-381j.

**24.** Jee YH, Emberson J, Jung KJ, et al. Cohort Profile: The Korean Cancer Prevention Study-II (KCPS-II) Biobank. *Int J Epidemiol.* Apr 1 2018;47(2):385-386f.

**25.** Bui AL, Katz R, Kestenbaum B, et al. Cystatin C and carotid intima-media thickness in asymptomatic adults: the Multi-Ethnic Study of Atherosclerosis (MESA). *Am J Kidney Dis.* Mar 2009;53(3):389-398.

**26.** Hillege HL, Fidler V, Diercks GF, et al. Urinary albumin excretion predicts cardiovascular and noncardiovascular mortality in general population. *Circulation.* Oct 1 2002;106(14):1777-1782.

**27.** Jassal SK, Kritz-Silverstein D, Barrett-Connor E. A prospective study of albuminuria and cognitive function in older adults: the Rancho Bernardo study. *Am J Epidemiol.* Feb 1 2010;171(3):277-286.

**28.** Kovesdy CP, Norris KC, Boulware LE, et al. Association of Race With Mortality and Cardiovascular Events in a Large Cohort of US Veterans. *Circulation.* Oct 20 2015;132(16):1538-1548.

**29.** Wong CW, Lamoureux EL, Cheng CY, et al. Increased Burden of Vision Impairment and Eye Diseases in Persons with Chronic Kidney Disease - A Population-Based Study. *EBioMedicine.* Mar 2016;5:193-197.

**30.** Tollitt J, Odudu A, Flanagan E, Chinnadurai R, Smith C, Kalra PA. Impact of prior stroke on major clinical outcome in chronic kidney disease: the Salford kidney cohort study. *BMC Nephrol.* Nov 27 2019;20(1):432.

**31.** Wen CP, Cheng TY, Tsai MK, et al. All-cause mortality attributable to chronic kidney disease: a prospective cohort study based on 462 293 adults in Taiwan. *Lancet.* Jun 28 2008;371(9631):2173-2182.

**32.** Bycroft C, Freeman C, Petkova D, et al. The UK Biobank resource with deep phenotyping and genomic data. *Nature.* Oct 2018;562(7726):203-209.

**33.** Nerpin E, Ingelsson E, Riserus U, et al. The combined contribution of albuminuria and glomerular filtration rate to the prediction of cardiovascular mortality in elderly men. *Nephrol Dial Transplant.* Sep 2011;26(9):2820-2827.
